# Agreement Between Tear Film Tests Performed by Novice Examiners

**DOI:** 10.1007/s44402-026-00152-x

**Published:** 2026-07-27

**Authors:** Miriam Casares-Lopez, Pilar Granados-Delgado, Francesco Martino, Miriam Carrillo-Pulido, Rosario G. Anera, Sonia Ortiz-Peregrina

**Affiliations:** Department of Optics, Faculty of Sciences, Laboratory of Vision Sciences and Applications, University of Granada, Granada, Spain

**Keywords:** Non-invasive tear break-up time, Novice examiners, Ocular surface disease index, Tear break-up time, Tear film

## Abstract

**Purpose:**

To explore the agreement among tear break-up time measurements performed by novice examiners using manual non-invasive tear break-up time (mNIBUT), automated non-invasive tear break-up time (aNIBUT) and fluorescein tear break-up time (FBUT), and to examine the association between tear film metrics and symptoms.

**Methods:**

A total of 61 volunteers (44 females) with a mean age of 21.5 ± 2.7 years underwent tear-film assessment performed by novice examiners (undergraduate students). The tear volume (Schirmer test), tear film stability by mNIBUT, aNIBUT and FBUT, along with lipid layer pattern, were assessed. Symptoms associated with tear-film quality and ocular surface discomfort were evaluated using the Ocular Surface Disease Index (OSDI) questionnaire.

**Results:**

Normal values were seen in 88.5%, 93.4%, 73.8% and 39.3% of participants using the Schirmer test, FBUT, mNIBUT and aNIBUT, respectively. The intraclass correlation coefficient (ICC) for tear break-up measurements indicated poor reliability between tests, except when comparing mNIBUT and FBUT (ICC = 0.67 and 0.60 for averaged and first measurements, respectively). Bland–Altman analysis showed that the 95% limits of agreement for mNIBUT-aNIBUT and FBUT-aNIBUT were wider than those for FBUT-mNIBUT. The lack of agreement did not change when averaging the three measurements. Results from the OSDI indicated that most participants did not report symptoms. No significant correlations were observed between the OSDI total score and any of the tear metrics (*p* > 0.05). The aNIBUT demonstrated the best agreement with symptoms in this young and largely asymptomatic population.

**Conclusions:**

Tear break-up time measurements obtained by novice examiners showed limited reliability and agreement when comparing traditional methods (FBUT and mNIBUT) and the aNIBUT. Examiner-related variability may influence the assessment of tear-film stability, although causal inferences regarding examiner experience cannot be established from this study design. Repeating measurements did not improve the reliability and agreement between tests when performed by novice examiners.

Key Points

- Automated tear film testing is no substitute for clinical judgement, because its results may differ from manual measurements made by novice examiners.
- Repeating tear film measurements does not necessarily improve consistency when examiners have limited experience, highlighting the importance of training and careful interpretation.
- Assessment of dry eye should combine clinical tests with patient-reported symptoms, since neither objective measurements nor symptoms alone provide a complete picture.

## Introduction

Evaluation of the tear film is a cornerstone in the diagnosis and management of ocular surface disorders, particularly dry eye disease (DED). Among the numerous clinical tests available, traditional methods such as the Schirmer test, introduced by Otto Schirmer in 1903 to estimate aqueous tear secretion and the fluorescein tear break-up time (FBUT) remain the most widely used. They are still considered key diagnostic criteria according to the *Tear Film and Ocular Surface Society Dry Eye Workshop III (TFOS DEWS III)* [1, 2]. Despite the emergence of advanced imaging-based devices, these classic tests continue to be the primary tools for evaluating tear production and stability in both clinical and research contexts [3, 4].

However, traditional techniques for assessing tear-film quality are inherently dependent on the examiner’s skill and subjective interpretation, which may compromise the accuracy and repeatability of the results. Even when performed by experienced clinicians, FBUT and Schirmer measurements show considerable intra- and inter-observer variability [5–7]. Furthermore, the use of fluorescein itself can destabilise the tear film and shorten the break-up time, thus influencing the reliability of the measurement [8, 9]. These limitations underscore the need for objective and reproducible alternatives that minimise human bias.

In the last 10 years, significant technological advances have led to the development of automated, image-based approaches, such as the non-invasive break-up time (NIBUT) obtained automatically with a corneal topographer. These instruments offer objective quantification of tear-film parameters and enable a more standardised evaluation of the ocular surface [10–12]. Nevertheless, the literature remains inconclusive regarding the agreement between automated and traditional techniques. While some authors have reported acceptable correlations between manual NIBUT and FBUT [13], others have demonstrated significant differences in the obtained values, suggesting that the two methods are not interchangeable [14–16].

This aspect gains particular importance considering that tear-film evaluations are often conducted by less experienced examiners, such as early-career clinicians with limited expertise in ocular surface assessment. Recent national data indicate that 407 new optometrists and around 300 new ophthalmologists enter the Spanish labour market each year, representing about 2–3% of the total eye-care workforce (Instituto Nacional de Estadística [INE], 2025; Ministerio de Sanidad, 2025). Given this continuous influx of novice professionals, understanding how examiner experience influences the reliability and agreement of tear-film tests is essential for ensuring diagnostic consistency.

The relationship between tear metrics and symptomatology is another key aspect in the diagnosis of tear-related problems. Assessing symptoms is essential in DED because they are often the patient’s first and most clinically meaningful manifestation of the disorder, affecting both comfort and visual function. Thus, it is important to consider the symptoms in order to study the real impact on the patient’s quality of life [17]. The discrepancy observed between signs and symptoms reflects the multifactorial nature of DED and emphasises the need for multimodal diagnostic approaches that integrate both objective measures and symptom-based assessments. This lack of agreement between signs and symptoms is due to different methodological, statistical and pathophysiological reasons [2]. Although this discrepancy is observed even when the tests are performed by experienced examiners [18, 19], analysing the agreement between signs and symptoms may identify the extent to which the subjective nature of tear tests and the methods undertaken influence this relationship.

Thus, the aim of the present study was to analyse the agreement among three tear break-up time methods (manual non-invasive break-up time (mNIBUT), automated non-invasive break-up time (aNIBUT) and FBUT), as well as the association between signs and symptoms for different tear metrics performed by examiners with limited experience.

## Methods

### Participants

A total of 61 volunteers (44 females and 17 males) took part in the study (mean age 21.5 ± 2.7 years). Prior to the examination, participants provided written informed consent in accordance with the Helsinki Declaration. Exclusion criteria included any ocular pathology, pregnancy or current use of pharmacological treatment that could affect visual function. Dry-eye condition was not an exclusion criterion. Participants were told not to wear contact lenses for at least 1 day and to have slept for at least 6 h the night before the trial. All the procedures described in the study were approved prospectively by the University of Granada Ethics Research Committee (2610/CEIH/2022).

### Tear-Film Assessment

#### Tear Volume

For tear-film assessment, one eye was selected at random. First, tear production was assessed by means of the Schirmer I test (without anesthesia) using I-DEW tear strips (Product code MIPL/A1; Madhu Instruments Pvt.; madhuinstruments.com). The Schirmer test provides an indirect but reliable estimation of the basal and reflex aqueous tear secretion and remains one of the most widely used and validated methods for assessing *tear volume in clinical and research settings* [1, 20]*. The* test consists of a 35 mm paper strip. The strip was carefully placed in the temporal third of the lower conjunctival sac. After 5 min, the strip was removed and the length of the moistened portion recorded in millimetres.

#### Tear Stability

The non-invasive tear break-up time was assessed both manually (mNIBUT) and automatically (aNIBUT) using the corneal topographer Topcon CA-800 Corneal Analyzer (model CA-800; VISIA Imaging S.r.I.; visiaimaging.com). The topographer has 24 rings that measure up to 6144 points of the eye’s surface. This device allows tear break-up to be assessed from 5% of the corneal surface during a normal inter-blink interval. The Topcon CA-800 has demonstrated good repeatability in contact lens users and has been used for the assessment of both manual and automated NIBUT [21, 22]. Since the experience of the examiner does not influence aNIBUT, only one measurement was obtained for this parameter, in order not to alter the tear film stability, as recommended by other authors [23, 24]. For mNIBUT, three measurements were taken consecutively.

FBUT was measured with a Topcon SL-D4 slit lamp biomicroscope (topconhealthcare.com) set at 16X, cobalt blue light, and a yellow filter (Live experience yellow filter; Conóptica S.L.; conoptica.es). A drop of saline solution was instilled on an I-DEW FLO fluorescein sodium strip (product code MIPL/A4; Madhu Instruments Pvt. Ltd; madhuinstruments.com) impregnated with 1 mg of sodium fluorescein, and the strip was shaken to remove excess fluorescein. After that, the fluorescein was applied to the upper bulbar conjunctiva, and participants were asked to blink for the fluorescein to be distributed evenly over the ocular surface. Participants were then instructed to blink twice, and after the second blink, to hold their blink for the measurements. Three FBUT measurements were taken consecutively.

#### Lipid Layer Thickness

The thickness of the lipid layer was tested with Keeler Tearscope-Plus (catalogue no. 2413-P-2004; Keeler Ltd; keelerglobal.com) attached to the Topcon SL-D4 slit lamp for better observation. Magnification was set at 20× and the brightest illumination level provided by the Tearscope was used. This device enables the direct observation of the colour interference pattern resulting from the reflection of white light in the tear film layers. The observed lipid layer pattern (LLP) was classified according to Guillon’s Clinical scheme, i.e., open meshwork, closed meshwork, wave, amorphous and colour fringe [25].

#### Ocular Surface Disease Index (OSDI)

To assess the symptoms associated with tear-film quality and ocular surface discomfort subjectively, participants completed the Ocular Surface Disease Index (OSDI) questionnaire [17]. The OSDI is a validated and widely used instrument designed to quantify the severity of dry-eye symptoms and their impact on vision-related functioning [17]. It consists of 12 items grouped into three subscales: (1) ocular symptoms (photophobia, grittiness and pain), (2) visual function (limitations in reading, computer use or driving) and (3) environmental triggers (wind, low humidity or air conditioning). Each item is rated from 0 (‘none of the time’) to 4 (‘all of the time’) and the total score is calculated as follows:

$${\rm{OSDI}}\; {\rm{score}}=\frac{({\rm{sum}} \;{\rm{of}} \;{\rm{scores}}) \times 25}{{\rm{number}} \;{\rm{of}} \;{\rm{questions}} \;{\rm{answered}}}$$

This yields a final score ranging from 0 to 100, where higher scores indicate greater disability. Scores from 0 to 12 correspond to normal eyes [20, 26]. This allowed concordance between signs and symptoms to be established as follows:

- Concordance was assumed when signs and symptoms agreed (i.e., both signs and symptoms or no signs or symptoms). If there were symptoms (e.g., OSDI > 12) and signs (aNIBUT/mNIBUT <10 s, FBUT < 5 s, Schirmer <10 mm/5 min), ‘positive agreement’ was considered. If there were no symptoms (OSDI ≤12) and no signs (aNIBUT/mNIBUT ≥10 s, FBUT ≥ 5 s, Schirmer ≥ 10 mm/5 min), then ‘negative agreement’ was considered.
- Non-concordance was assumed when (1) signs and symptoms did not agree, i.e., when there were symptoms (OSDI score >12) but no signs (mNIBUT/aNIBUT ≥ 10 s, FBUT ≥ 5 s, Schirmer ≥ 10 mm/5 min) or (2) there were no symptoms (OSDI score ≤12), but there were signs (mNIBUT/aNIBUT <10 s, FBUT < 5 s, Schirmer <10 mm/5 min).

#### Procedure

Measurements were all carried out by undergraduate students from the University of Granada at the laboratories of Optics (Faculty of Sciences, Granada, Spain). The students had previously worked in the optometry and contact lens laboratories at this university for more than 100 h, but they had never worked in an optometry office or laboratory outside the university setting. A total of 50 different individuals performed the measurements in order to maintain the lack of experience. Nevertheless, these novice examiners received 30 min training before performing the measurements and were supervised by experienced practitioners. The order of testing different tear-related parameters was randomised.

### Statistical Analysis

IBM SPSS V.26 software (ibm.com) was used to perform the statistical analysis. The normality of the data was tested with the Kolmogorov–Smirnov test. First, to decide whether one eye or both eyes should be analysed, a Spearman correlation test was performed. Mean values ± standard deviations (SD) were reported for normally distributed data and medians and interquartile ranges (IQR), along with the minimum and maximum values (range) for non-normal data. For the OSDI score, the mean ± SD and the IQR were provided. The association between signs and symptoms was explored with the Spearman correlation test, providing the correlation coefficient (rho) and *p*-value. Descriptives from the mean OSDI score for participants with normal and non-normal tear film values were provided, as well as the percentage and descriptives of participants with concordance or non-concordance between signs and symptoms. The reliability and agreement between the tear break-up tests (mNIBUT, aNIBUT and FBUT) were tested, while LLP results, which presented a qualitative character (thus, LLP was considered as a categorical variable), were only included as an additional descriptive metric to simplify the analysis, as well as the results and the information provided. Intraclass correlation coefficients (ICC) were used to assess relative reliability between tests, reflecting the consistency of the ranking of subjects across measurements. The ICC was calculated for each pair of methods (mNIBUT-FBUT; FBUT-aNIBUT and mNIBUT-aNIBUT), along with the 95% confidence intervals (95% CI). The absolute agreement and interchangeability between measurements were analysed using Bland–Altman analysis [27]. For each pairwise comparison (mNIBUT-FBUT; FBUT-aNIBUT and mNIBUT-aNIBUT), the mean difference, limits of agreement (LoA) and the 95% CI were reported, as well as the regression coefficient (*r*^2^) and the *p*-value. The significance level was set at *p* < 0.05 for all tests. Finally, G*Power 3 software (psychologie.hhu.de/arbeitsgruppen/allgemeine-psychologie-und-arbeitspsychologie/gpower) was used to determine the minimum detectable correlation between the OSDI score and tear-film metrics. Under these assumptions, the study was able to detect correlations of approximately *ρ* ≥ 0.35; therefore, the study was adequately powered to detect moderate associations. Since data from the two eyes were correlated (*p* = 0.10 for aNIBUT; *p* < 0.001 for mNIBUT and FBUT), one eye was selected at random from each participant for the statistical analysis [28].

## Results

### Descriptive Statistics

Results of the tear film assessment are represented in Table 1. According to the DEWS III, normal values (cutoff values of dry eye) for the FBUT are ≥ 5 s, and ≥ 10 s for the NIBUT and aNIBUT [2]. Cutoff values for the Schirmer test are ≥ 10 mm/5 min [23, 24]. The percentage of participants with normal values was: 88.5%, 93.4%, 73.8% and 39.3% for the Schirmer test, FBUT (averaged), mNIBUT (averaged) and aNIBUT, respectively. No significant differences were observed between the first measurement and the averaged FBUT (*z* = −0.52; *p* = 0.60) and mNIBUT (*z* = −0.09; *p* = 0.93). Additionally, the standard errors for the FBUT and mNIBUT were quite similar when comparing the first measurement and the average (1.38 vs 1.29, respectively, for the FBUT and 1.18 vs 1.20 for the mNIBUT).

**Table 1:** Median and IQR for the tear parameters (Schirmer and tear break-up time measured with FBUT, mNIBUT and aNIBUT) and the range (minimum and maximum).

		Median (IQR)	Range
Schirmer (mm/5 min)		25.00 (15.00–31.00)	4.00–35.00
FBUT (s)	First measurement	13.35 (8.85–20.30)	2.00–60.00
	Averaged	13.98 (9.06–19.38)	4.00–60.00
mNIBUT (s)	First measurement	13.02 (9.50–16.17)	3.06–60.00
	Averaged	13.02 (9.50–16.17)	3.06–60.00
aNIBUT (s)		7.50 (2.80–14.76)	1.30–29.96
LLP		Frequency	%
Open meshwork		9	14.8
Closed meshwork		21	34.4
Wave		24	39.3
Amorphous		7	11.5
Colour fringe		–	–
Globular		–	–

*aNIBUT* automated non-invasive break-up time, *FBUT* fluorescein break-up time, *IQR* interquartile range, *LLP* lipid layer pattern, *mNIBUT* manual non-invasive break-up time, *TBUT* tear break-up time.

The results of the lipid layer (LLP) are also included.

As can be observed in Table 1, none of the participants presented a pattern corresponding to a thick lipid layer (colour fringe and globular); instead, most presented patterns corresponding to a normal or thin lipid layer (wave and close meshwork, respectively).

### Concordance Between Tear Break-up Time Measurements

#### First Measurement

The relative reliability between measurements was tested by calculating the 2-way mixed-effects, absolute agreement ICC. First, the three methods were considered for the reliability analysis, yielding the following results: ICC (3,1) = 0.33, 95% CI = (0.18–0.39), *p* < 0.001. Thus, ICC for the average measurements (0.33) indicated poor reliability from any of the methods. To explore which of the methods was responsible for this poor reliability, a two-by-two reliability analysis was performed, also providing the single-measure ICC for each pair of measurements. These results are shown in Table 2. As can be observed, the lower ICC was obtained when comparing the subjective methods (both FBUT and mNIBUT) with the objective technique (aNIBUT), indicating that the automated measurements were poorly correlated with traditional subjective measurements. The two tests that presented moderate reliability were the manual mNIBUT and FBUT (ICC = 0.60).

**Table 2:** Results from the reliability analysis for the first measurement, including intraclass correlation coefficient (with 95% confidence intervals and *p*-values) and Bland–Altman analysis (mean difference and SD, limits of agreement and 95% confidence intervals) comparing the three methods for measuring tear break-up time (mNIBUT, aNIBUT and FBUT), along with the linear regression (*R*^2^ and *p*-value).

		mNIBUT - aNIBUT	FBUT - mNIBUT	FBUT - aNIBUT
Reliability analysis	ICC (3,1) (95% CI)	0.00 (−0.22 to 0.23)	0.60 (0.42–0.74)	0.005 (−0.20 to 0.41)
	*p*-value	0.50	<0.001	0.48
Bland–Altman analysis	Mean difference (s) (SD)	4.50 (12.65)	1.47 (8.90)	5.96 (13.80)
	Upper LoA (s) (95% CI)	29.30 (34.91–23.68)	18.92 (22.87–14.97)	33.01 (39.13–26.89)
	Lower LoA (s) (95% CI)	−20.30 (−14.69 to −25.91)	−15.99 (−12.04 to −19.94)	−21.09 (−14.96 to −27.21)
Linear regression	*R*^2^	0.004	0.02	0.05
	*p*-value	0.63	0.13	0.09

*aNIBUT automated non-invasive break-up time, CI* confidence intervals, *FBUT* fluorescein break-up time, *ICC* intraclass correlation coefficient, *LoA* limits of agreement, *mNIBUT* manual non-invasive break-up time.

Bland–Altman analysis provided information regarding the absolute agreement and interchangeability between measurements (BUT, mNIBUT and aNIBUT). The mean difference between the measurements was provided, along with the LoA, calculated as: LoA = mean ± (SD × 1.96). The 95% CI of the LoA was calculated as $\sqrt{3{s}^{2}/n}$, where *s* is the standard deviation of the differences and *n* is the sample size [27]. These results are shown in Table 2.

**Fig. 1:**
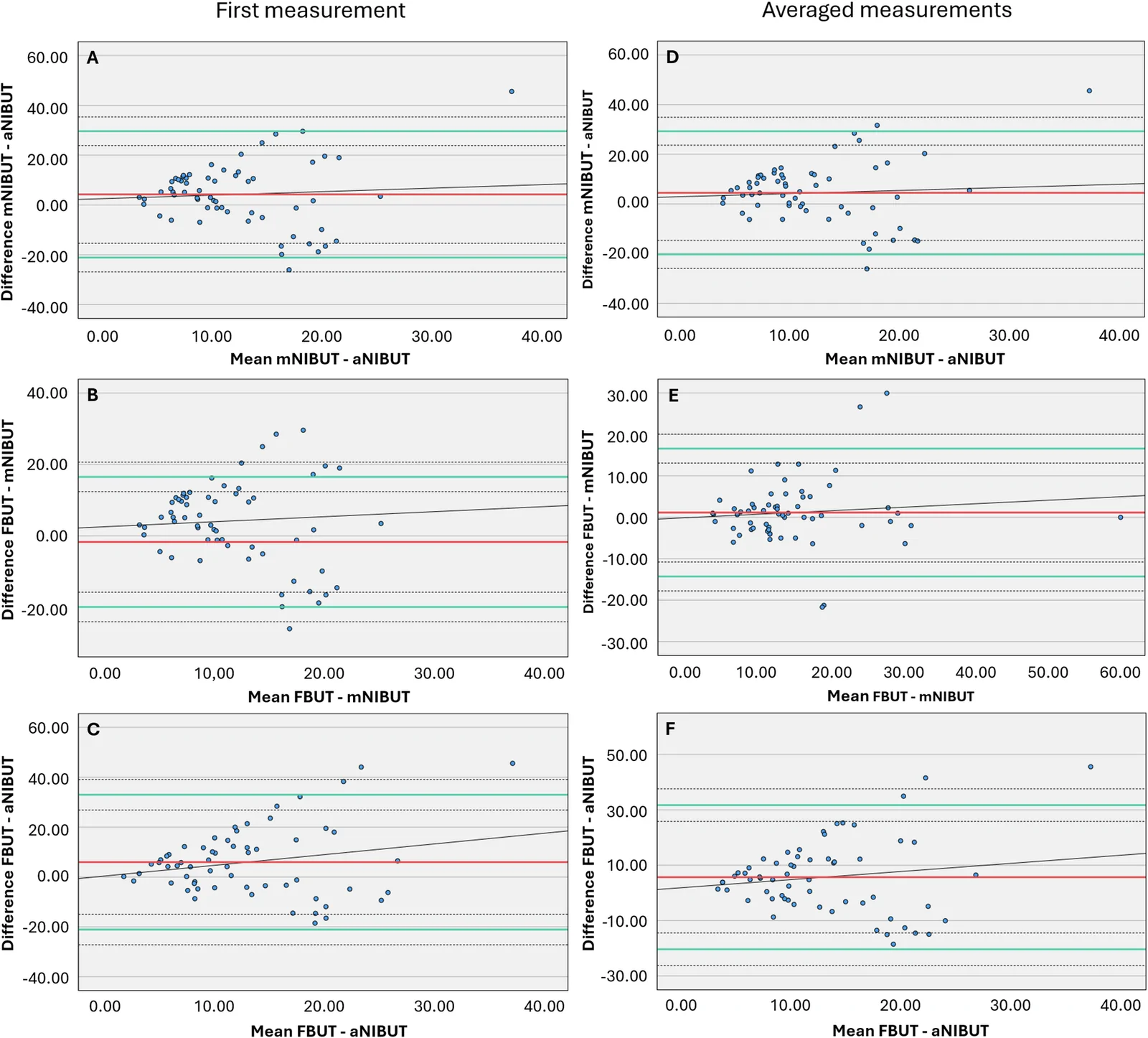
Bland–Altman plots from two-by-two comparisons of the three methods for measuring the tear break-up time. The mean (red line), the limits of agreement (green lines) and their 95% confidence intervals (dotted lines), as well as the regression line (solid black line) are indicated. **A** mNIBUT (manual non-invasive break-up time) vs. aNIBUT (automated non-invasive break-up time), first measurement. **B** FBUT (fluorescein break-up time) vs. mNIBUT, first measurement. **C** FBUT vs. aNIBUT, first measurement. **D** mNIBUT vs. aNIBUT, averaged measurements. **E** FBUT vs. mNIBUT, averaged measurements. **F** FBUT vs. aNIBUT, averaged measurements.

As can be observed in Table 2, the mean differences were relatively low, particularly for the FBUT-mNIBUT comparison. When comparing mNIBUT-aNIBUT and FBUT-aNIBUT, the mean differences were higher, but these values (4.50 and 5.97 s) are within the variability range provided by other authors when analysing the repeatability of clinical measurements of tear break-up time (both FBUT and mNIBUT) [7, 29]. The Bland–Altman analysis indicates that, although the mean bias was small, the 95% LoA were wide, suggesting that these methods are not interchangeable (Fig. 1). Particularly, for the mNIBUT-aNIBUT and FBUT-aNIBUT comparisons (Fig. 1A, C, respectively), the LoA were wider. When comparing FBUT-mNIBUT, the LoA were narrower.

Regression analysis (Table 2) revealed that the differences between the methods (mNIBUT-aNIBUT, FBUT-mNIBUT and FBUT-aNIBUT) were not significantly correlated with the values obtained for the tear break-up time (*R*^2^ = 0.004–0.05; *p* > 0.05), i.e., the agreement between methods does not change depending on whether the values of tear break-up time were lower or higher.

#### Averaged Measurements

The reliability of the averaged measurements was also assessed using the ICC, Bland–Altman analysis and linear regression, as shown in Table 3. For the ICC analysis, the results revealed moderate to poor reliability. Specifically, the ICC for the comparison between mNIBUT and aNIBUT was very low (0.005), with the confidence interval (95% CI) ranging from −0.22 to 0.24 and *p* = 0.48, indicating no significant agreement. In contrast, comparison between FBUT and mNIBUT showed a stronger ICC of 0.67 (95% CI = 0.51–0.79), *p* < 0.001, suggesting moderate reliability. However, the ICC for FBUT-aNIBUT remained low (ICC = −0.003, 95% CI = −0.21 to 0.22), *p* = 0.51, indicating poor agreement between these methods. And again, the Bland–Altman analysis showed that the 95% LoA were wide, particularly for mNIBUT-aNIBUT and FBUT-aNIBUT, revealing that these methods were not interchangeable (Fig. 1D, F).

**Table 3:** Results from the reliability analysis for the three averaged measurements, including intraclass correlation coefficient (with 95% confidence intervals and *p*-value) and Bland–Altman analysis (mean difference and SD, limits of agreement and 95% confidence intervals) comparing the three methods for measuring tear break-up time (mNIBUT, aNIBUT and FBUT), along with the linear regression (*R*^2^ and *p*-value).

		mNIBUT - aNIBUT	FBUT - mNIBUT	FBUT - aNIBUT
Reliability analysis	ICC (3,1) (95% CI)	0.005 (−0.22 to 0.24)	0.67 (0.51 – 0.79)	−0.003 (−0.21 to 0.22)
	*p*-value	0.48	<0.001	0.51
Bland–Altman analysis	Mean difference (s) (SD)	4.54 (12.72)	1.16 (7.87)	5.70 (13.28)
	Upper LoA (s) (95% CI)	29.464 (35.10–23.82)	16.59 (20.08–13.10)	31.74 (37.63–25.85)
	Lower LoA (s) (95% CI)	−20.38 (−14.74 to −26.02)	−14.27 (−10.78 to −17.76)	−20.34 (−14.44 to −26.23)
Linear regression	*R*^2^	0.009	0.02	0.006
	*p*-value	0.46	0.26	0.56

*aNIBUT automated non-invasive break-up time, CI* confidence intervals, *FBUT* fluorescein break-up time, *ICC* intraclass correlation coefficient, *LoA* limits of agreement, *mNIBUT* manual non-invasive break-up time, *SD* standard deviation.

Similarly, regression analysis considering the averaged measurements (Table 3) indicated that the agreement between methods did not change whether the values of tear break-up time were lower or higher (*R*^2^ = 0.006–0.02; *p* > 0.26).

#### Relationship Between Signs and Symptoms

OSDI results are shown in Table 4. The mean values for the different items indicate that most participants did not have dry eye symptoms or only had them some of the time. Thus, the mean total score (18.96 ± 11.56) revealed that most participants had normal eyes (0–12) or mild dry eye [20, 26]. Although the total OSDI score was higher for contact lens users (Mean = 20.96; SD = 11.09; *n* = 27) compared with non-users (Mean = 17.38; SD = 11.83; *n* = 34), no significant differences were found (*t*(59) = −1.21; *p* = 0.25).

**Table 4:** Results of the Ocular Surface Disease Index (OSDI) questionnaire, providing mean ± SD and the IQR for each item, each block (sub-total) and the total score calculated as: (sum of scores × 25)/number of questions answered.

Question	Mean ± SD	IQR
Have you experienced any of the following during the last week?		
1. Eyes that are sensitive to light?	0.97 ± 0.71	0.50–1.00
2. Eyes that feel gritty?	0.61 ± 0.71	0.0–1.00
3. Painful or sore eyes?	0.93 ± 0.68	0.50–1.00
4. Blurred vision?	0.67 ± 0.72	0.0–1.00
5. Poor vision?	0.48 ± 0.60	0.00–1.00
Sub-total	3.66 ± 2.44	2.00–5.50
Have problems with your eyes limited you in performing any of the following during the last week?		
6. Reading?	0.61 ± 0.82	0.0–1.00
7. Driving at night?	0.39 ± 0.67	0.00–1.00
8. Working with a computer or bank machine (automatic teller machine, ATM)?	0.85 ± 0.85	0.0–1.00
9. Watching television?	0.75 ± 0.79	0.00–1.00
Sub-total	2.61 ± 2.40	1.00–4.00
Have your eyes felt uncomfortable in any of the following situations during the last week?		
10. Windy conditions?	1.08 ± 0.78	1.0–2.00
11. Places or areas with low humidity (very dry)?	0.52 ± 0.79	0.0–1.00
12. Areas that are air-conditioned?	1.02 ± 0.99	0.00–2.00
Sub-total	2.62 ± 2.03	1.00–4.00
Total score	18.96 ± 11.56	8.33–25.00

*SD* standard deviation, *IQR* interquartile range.

The means and SD values of the OSDI questionnaire for normal and non-normal participants according to their tear test results are represented in Table 5. As can be observed, very small differences were observed between these two groups, particularly for mNIBT, aNIBUT and LLP. In the case of FBUT and Schirmer test, the mean OSDI score was slightly higher in the non-normal group, indicating more symptoms.

**Table 5:** Mean values and SD of the OSDI questionnaire for normal and non-normal participants according to their tear test results.

		Mean OSDI score ± SD
FBUT	Normal (*n* = 47)	18.66 ± 11.80
	Non-normal (*n* = 4)	23.26 ± 6.62
mNIBUT	Normal (*n* = 45)	19.51 ± 12.19
	Non-normal (*n* = 16)	17.42 ± 9.73
aNIBUT	Normal (*n* = 24)	18.73 ± 10.13
	Non-normal (*n* = 37)	19.12 ± 12.53
Schirmer	Normal (*n* = 54)	18.46 ± 11.76
	Non-normal (*n* = 7)	22.82 ± 9.68
LLP	Normal (*n* = 32)	18.98 ± 11.20
	Non-normal (*n* = 28)	19.02 ± 12.35

Note that normal LLP was considered when wave pattern or amorphous pattern were found.

*FBUT* fluorescein break-up time, *mNIBUT* manual non-invasive break-up time, *aNIBUT* automated non-invasive break-up time, *LLP* lipid layer pattern, *SD* standard deviation, *OSDI* Ocular Surface Disease Index.

**Fig. 2:**
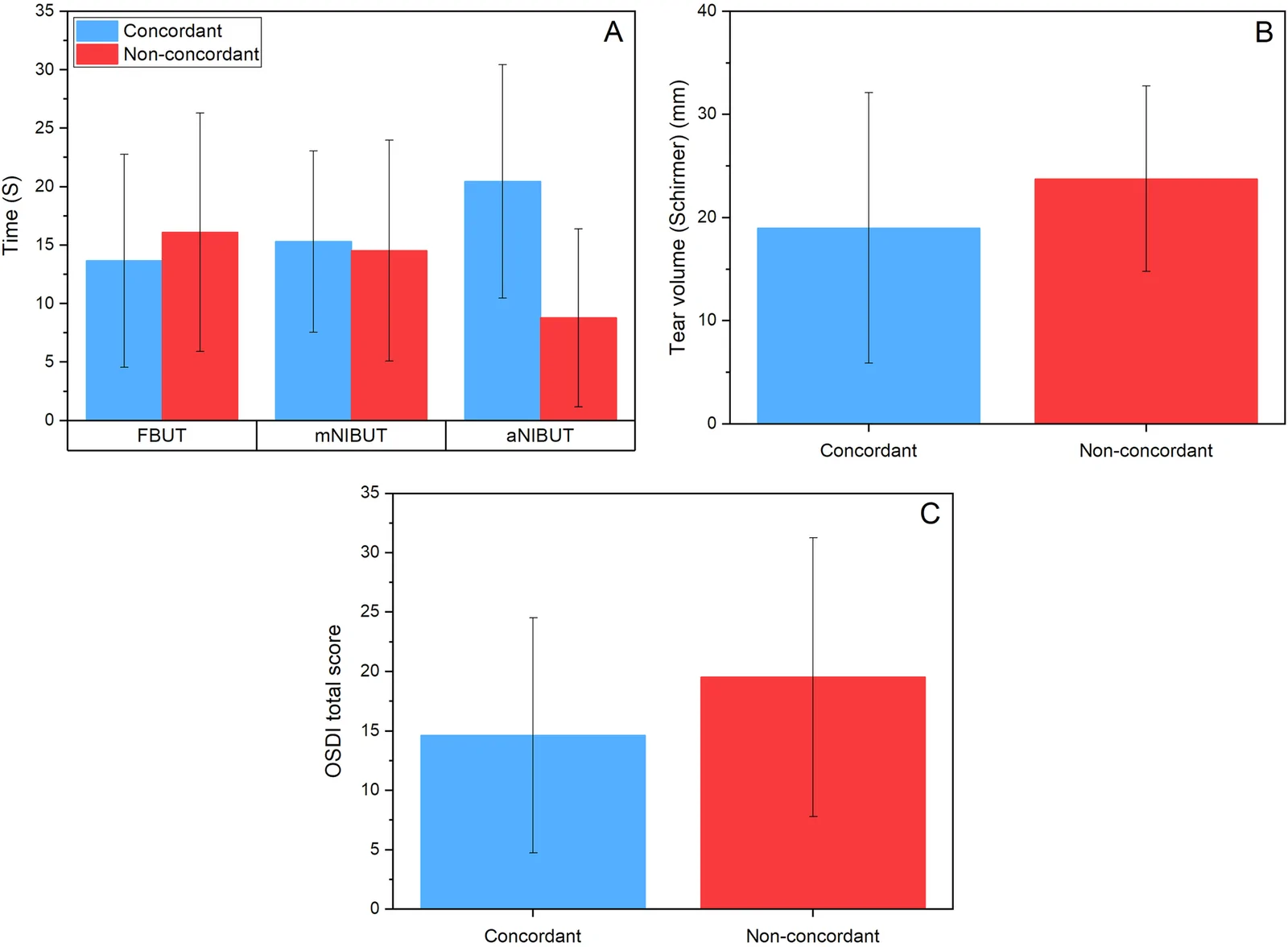
Comparison of the tear film metrics (FBUT, mNIBUT and aNIBUT (**A**), Schirmer test (**B**)) and Ocular Surface Disease Index (OSDI) total score (**C**) in participants with concordance (blue) and non-concordance (red) between signs and symptoms. FBUT fluorescein break-up time, mNIBUT manual non-invasive break-up time, aNIBUT automated non-invasive break-up time.

Additionally, no significant correlations were observed between the OSDI total score and the tear film metrics: Schirmer test, FBUT, mNIBUT and aNIBUT (*p* > 0.52). In fact, the results revealed signs and symptoms agreed for 41% participants in the case of the Schirmer test (31.2% negative agreement, 9.8% positive agreement), 45.9% for mNIBUT (26.2% negative agreement, 19.7% positive agreement), 39.3% for FBUT (32.8% negative agreement, 6.6% positive agreement) and 60.7% for aNIBUT (6% negative agreement, 31% positive agreement). Considering the four metrics, only 9.8% of participants (6 out of 61) presented concordance among the metrics and the total OSDI score (8.2% negative agreement, 1.6% positive agreement). The mean results and SD of the different tear metrics and OSDI score in the two groups are shown in Fig. 2.

## Discussion

This study aimed to explore the agreement between tear break-up time obtained by undergraduate students using manual methods vs automatically with a corneal topographer, as well as the relationship between different tear film metrics and symptoms. Traditional measurements comprise the assessment of both invasive (FBUT) and non-invasive (NIBUT) break-up times, tear volume using the Schirmer test and LLP. For most of the participants, the results obtained from tear film metrics were typical of healthy eyes, and the OSDI score indicated no symptoms, except in the case of the aNIBUT findings, which were within the range for non-normal (dry) eyes in most cases. The reliability of the FBUT and mNIBUT tests did not improve when considering the average instead of the first measurement. These results contrast with previous works that reported an improvement in the reliability of TBUT when measurements were taken by experienced observers [7]. However, this previous study included only one examiner, so these contradictory findings may reflect the examiner-related variability in the present study, which included 50 different examiners.

On the other hand, the ICC analysis, which reflects the extent to which the different methods preserved the ranking of participants, showed larger differences when comparing FBUT and aNIBUT, as well as when comparing mNIBUT and aNIBUT. Also, it should be noted that this lack of reliability between tests did not change when the averaged measurements were considered. A previous study that compared automated and manual NIBUT obtained with the same device obtained a lower difference of only 0.3 s, along with a good correlation between the two measurements [12]. These findings indicate the limited reliability of tear break-up time results in this novice-examiner setting. Although the lack of experience of the examiners must be considered, it should be noted that examiner heterogeneity and procedural variability may also have influenced the results. In fact, the measurements performed in the present study showed poor reliability, particularly when comparing the traditional methods for assessing tear stability (FBUT and mNIBUT) with the aNIBUT; however, when comparing FBUT and mNIBUT, good reliability was obtained. This finding was striking, since most previous studies that compared traditional and automated TBUT obtained by experienced professionals reported good reliability when comparing automated vs manual findings measured with the same device [12], but lower reliability (ICC) when comparing FBUT and NIBUT [13]. However, the present results indicate that aNIBUT was not significantly correlated with mNIBUT and FBUT. Since the Topcon CA-800 records the aNIBUT as the moment when 5% of the tear film breaks, one possible explanation is delayed recognition of tear break-up by manual observation. This hypothesis is supported by the lower mean values obtained for the aNIBUT, compared with mNIBUT and the FBUT, contrary to the results reported by others, who found that the aNIBUT obtained by experienced examiners using a topographer was longer than the FBUT [13]. Although non-invasive methods avoid the potential tear-film disturbance induced by fluorescein instillation, this does not necessarily imply that automated NIBUT values will be longer than FBUT values in all settings. Previous studies have reported variable findings, probably reflecting differences in device algorithms, break-up detection thresholds, measurement protocols, fluorescein volume and study populations [2, 12, 16]. Therefore, the lower aNIBUT values observed in the present study should be interpreted cautiously and may reflect both device-specific detection criteria and the novice examiners, rather than the effect of fluorescein alone. However, lack of experience may not be the only reason to explain the lower aNIBUT values, since it has been reported that the Topcon CA-800 underestimates the aNIBUT compared to the subjective technique, even when measurements are conducted by experienced examiners [21]. Also, as commented before, the inclusion of 50 undergraduate examiners introduces inter-examiner variability inherent to the study design, which may have contributed to the observed differences in reliability between tests.

The Bland–Altman analysis was performed to explore whether the three metrics (FBUT, mNIBUT and aNIBUT) were interchangeable, i.e., the absolute agreement between tests. First, acceptable interchangeability should be judged by comparing the Bland–Altman LoA against a priori clinical tolerances. Tolerances can be anchored to (1) within-method repeatability and (2) diagnostic decision thresholds. Prior work reported within-method repeatability for FBUT with LoA of approximately ±5.8 s, suggesting that a between-method LoA wider than about ±6 s is unlikely to be clinically acceptable for individual-level substitution [7]. Moreover, because optimal diagnostic cut-points for FBUT have been estimated around 5.3–6.0 s, a between-method LoA > ±3–5 s may induce threshold-crossing misclassification near the decision boundary [30]. In the present dataset, the LoA for FBUT–mNIBUT, and especially for comparisons involving aNIBUT, were substantially wider, considering both the first measurement and averaged TBUT, consistent with recent reports showing LoA of the order of 26–31 s across devices and methods and concluding non-interchangeability [10, 16]. Therefore, while mean differences were small, the observed LoA exceeded plausible clinical tolerances. These results indicate that small mean differences do not imply clinical interchangeability when LoA are wide; substituting one TBUT modality for another in routine diagnostics would not be appropriate, particularly in settings involving novice examiners.

Other authors have obtained similar results for TBUT values acquired by experienced eye care practitioners. A weak association between NIBUT and FBUT has also been observed in previous studies [16, 31]. Similarly, other authors reported that automated NIBUT values obtained with different corneal topographers, e.g., Keratograph 5M (OCULUS, Optikgeräte GmbH; oculus.de) IDRA (SBM Sistemi S.r.l.; sbmsistemi.com) or Medmont E300 (Medmont International Pty Ltdl; medmont.com.au) were not interchangeable [16, 32]. These findings suggest that considering a cut-off value of 10s may not be appropriate for all TBUT metrics and measurement devices. Furthermore, the results from the agreement analysis show that considering the average of three measurements does not improve agreement when TBUT tests are performed by novice examiners. In this regard, previous work has concluded that the correlation between manual NIBUT and FBUT increased with repeated measurements [33]. Thus, it seems that the lack of operator experience influenced this aspect.

Regarding the reported symptoms, assessed with the OSDI questionnaire, the results showed no significant differences between normal and non-normal eyes, classified according to TBUT, volume (Schirmer test) and lipid layer pattern (LLP). Also, symptoms were not related to ocular surface disturbances, as assessed with the OSDI questionnaire and the results obtained in tear-film metrics, such as FBUT, mNIBUT and aNIBUT, or the tear volume assessed with the Schirmer test. Other authors have reported a weak association between signs and symptoms [20, 34–36]. This observation is due to different factors. First, symptoms seem to be more associated with personal and environmental factors than with tear physiology; second, alterations of the mucin layer, which are not usually addressed, may also be responsible for symptoms, inflammation and hyperosmolarity; third, symptom measures may assess particular properties of the disease (e.g., grittiness or fatigue), which may not be related to measurable signs and finally, psychological and sensorial adaptation modifies the perception of ocular discomfort [34, 36]. Compared with the results of the present study, other authors found a higher percentage of sign-symptom concordance for the Schirmer and BUT tests [34]. On the other hand, it should be noted that aNIBUT was the only variable that did not lie in the normal range for most participants and that most cases of sign-symptom concordance were positive, which may be explained by the fact that this test (the aNIBUT performed with Topcon CA-800) underestimates NIBUT values, as mentioned above.

Thus, two ideas are drawn from these results. First, aNIBUT showed the highest sign-symptom concordance among the metrics evaluated. However, this finding should not be interpreted as evidence that aNIBUT is the best predictor of symptoms, since the analysis was based on a small concordant subgroup and no formal predictive modelling was performed. Second, contrary to other works, measurements were performed by a high number of inexperienced examiners. The fact that a lower percentage of participants showed agreement between signs and symptoms compared to previous works highlights the need for cautious interpretation of tear-film assessments in training settings, particularly when applying tests that are more subjective in nature. Although automated and objective tear-film measurements do not solve the discrepancy between signs and symptoms [20, 37], these results suggest that early exposure to automated diagnostic systems, such as corneal topographers, may help students develop a better understanding of tear film dynamics and variability. However, the interpretation of automated findings still requires clinical judgment. As automated and more objective metrics become increasingly prevalent in ocular surface assessment, future training programmes should focus on integrating technology with traditional clinical skills to ensure competent interpretation of both subjective and objective findings [11, 13]. The weak association between signs and symptoms found in the present study also highlights the complexity of ocular surface disorders. This discrepancy underscores the necessity of integrating both objective and subjective assessments when diagnosing DED. Therefore, clinicians should avoid relying solely on TBUT or Schirmer results to make clinical decisions, particularly when the measurements are performed by less experienced examiners. The inclusion of validated questionnaires, such as the OSDI, and the use of multimodal diagnostic algorithms (combining non-invasive, automated and subjective methods) are likely to improve the accuracy of clinical evaluations [20, 36].

In the present study, the median Schirmer I value was relatively high (25 mm/5 min), with several participants approaching the maximum wetting length of the strip. Since the test was performed without topical anesthesia, these values should be interpreted as an estimate of total aqueous secretion under stimulated conditions rather than as a pure measure of basal tear production. The insertion of the paper strip may elicit reflex tearing, particularly in young participants with preserved lacrimal gland function, and this response may mask subtle differences in resting tear volume. Therefore, in this sample, the Schirmer test was useful mainly to rule out clinically relevant aqueous-deficient dry eye, rather than to explain ocular symptoms or tear-film instability. This interpretation is consistent with the absence of a significant association between Schirmer values and OSDI scores noted in the present study. Previous reports have shown that Schirmer test results are variable, may have limited reproducibility [2, 7] and often show weak or inconsistent associations with dry-eye symptoms and other ocular surface signs [38]. Thus, high Schirmer values in this young and mostly asymptomatic cohort should not be interpreted as evidence of superior tear-film homeostasis, but rather as a possible consequence of reflex tearing and the limited usefulness of Schirmer I for characterising tear-film alterations outside the context of aqueous-deficient dry eye. These findings support the need to interpret Schirmer results together with tear-film stability measures, ocular surface findings and symptom questionnaires.

However, several limitations should be considered when interpreting these findings. First, no formal a priori sample size calculation was performed. This was a first exploratory study designed to examine the agreement between tear break-up time methods when performed by novice examiners and therefore, the sample was based on the availability of eligible volunteers. The study sample was composed of young volunteers, most of whom were asymptomatic or only mildly symptomatic and predominantly had tear-film values within normal ranges. Therefore, the results should not be extrapolated directly to older populations or to patients with clinically established DED. In addition, the sign-symptom concordance analysis was exploratory and limited by the small number of participants showing complete concordance across metrics and symptoms. Future studies including larger and clinically more heterogeneous samples, together with formal predictive modelling, are needed to determine whether automated NIBUT contributes independently to symptom classification or dry-eye diagnosis. Another aspect that should be considered is the potential influence of test order and tear film perturbation. Although the order of tear film assessments was randomised to minimise systematic order effects, this approach does not completely eliminate possible carry-over effects between tests. This issue may have contributed to the variability observed between methods, particularly to the wide LoA found in the Bland–Altman analyses and to the poor agreement between aNIBUT and the traditional tear break-up time measurements. However, since the order was randomised, this effect would be expected to increase random variability rather than introduce a consistent directional bias.

## Conclusions

This study demonstrated that TBUT measurements obtained by novice examiners showed limited reliability when comparing traditional methods (FBUT and mNIBUT) with aNIBUT. Automated NIBUT values were lower than those obtained with mNIBUT and FBUT, and showed poor reliability with both subjective techniques, indicating that aNIBUT should not be considered interchangeable with traditional TBUT methods in this context. These results indicate that although non-invasive methods avoid the potential tear-film disturbance induced by fluorescein instillation, this does not necessarily imply that aNIBUT will be longer than FBUT values in all settings. Results also revealed that repeated measurements of TBUT, when performed by novice examiners, may not improve reliability and the agreement between tests. Moreover, the weak association observed between clinical signs and subjective symptoms reinforces the multifactorial nature of dry-eye disease and the need to combine both objective and subjective approaches for an accurate diagnosis, particularly when tear assessment is conducted by inexperienced examiners.

These findings highlight that for novice examiners, automated keratography does not yet ensure interchangeability with traditional TBUT methods and that early training in both manual observation and digital interpretation is essential. Despite these limitations, aNIBUT may be useful as an adjunctive tool in training settings, but its outputs still require clinical interpretation and should not be used interchangeably with manual methods. On the other hand, considering the lack of agreement among current measures, further research would be valuable to determine which tests, or combinations of tests, best predict disease progression and long-term ocular surface damage to improve the assessment of DED and interpretation of diagnostic results.

## Data Availability

The data that support the findings of this study are available from the corresponding author upon reasonable request.

## Statements and Declarations

The study was conducted in accordance with the Declaration of Helsinki and approved by the University of Granada Ethics Research Committee (2610/CEIH/2022) for studies involving humans. Informed consent was obtained from all subjects involved in the study.
